# Open reduction and plate fixation versus sling in treatment of mid-shaft fractures of clavicle: A prospective randomized study protocol

**DOI:** 10.1097/MD.0000000000023910

**Published:** 2021-01-29

**Authors:** Guanggao Li, Junjie Liao, Wencai Su

**Affiliations:** a Department of Orthopedics, Yanbian University Hospital, Jilin, 133000; b Department of Radiology, Huizhou Central Municipal Hospital, Guangdong, 516001; c Department of Orthopedics, Wuzhong People's Hospital, Ningxia, 751100, China.

**Keywords:** mid-shaft clavicle fractures, open reduction and internal fixation, randomized, study protocol

## Abstract

**Background:**

For this prospective randomized clinical trial, it is implemented for the comparison of the outcomes of open reduction and internal fixation versus the sling treatment for mid-shaft clavicle fractures.

**Methods:**

We will evaluate the eligible patients diagnosed with the mid-shaft clavicle fractures in our hospital from December 2020 to December 2021. The outcomes acquired were reported in accordance with the guidelines of Consolidated Standards of Reporting Trials (CONSORT). Approval for this prospective randomized clinical trial was obtained from the institution alethics review committee of Wuzhong People's Hospital. The criteria for inclusion included: one third of clavicle fracture with at least 1 axial width displacement (Robinson type 2B2 or 2B1); agreed to take part in our research; over 18 years of age; patients with isolated clavicle fracture. While the criteria for exclusion contained: the medial or lateral segment clavicle fracture; open fracture; injuries related to neurovascular; over 21 days of changes from accident. The primary outcome was described as the evidence of nonunion at 1 year, which was defined as the intact bone bridge without X-ray fracture after more than 6 months. The secondary outcomes included the function of arm, overall health, adverse events, the satisfaction related to appearance, and pain.

**Conclusions:**

It was hypothesized that in the case of clavicular comminuted mid-shaft fracture, for the open reduction and internal fixation, its nonunion rate was lower and satisfaction rate was higher, although there were a variety of complications.

**Trial registration:**

This study protocol was registered in Research Registry (researchregistry6295).

## 1 Introduction

Mid-shaft fractures of clavicle are common injury, accounting for 81% of all clavicular fractures, and are often accompanied by displacement.^[1]^ In the past, non-surgical treatment has been preferred for axial clavicular fractures because of the extremely low rate of bone nonunion, even in the case of significant displacement.^[2,3]^ However, a number of scholars have recently found that nonoperative displaced fractures have a higher rate of nonunion than previously reported. At present, a consensus has been reached on the non-surgical treatment of undisplaced central clavicle fractures. However, the optimal treatment for displaced central clavicle fractures is controversial.^[4–7]^

For the treatment of clavicle fracture, its target is to prevent disability and restore the function of upper limb. In 2007, the Canadian Orthopaedic Trauma Society implemented the randomized clinical trial (RCT) and summarized that the surgical treatment of displaced fractures of the clavicle decreased the incidence rate of nonunion and malunion and enhanced the functional results compared to the non-surgical treatment as assessed in 1-year follow-up.^[8]^ In contrast, Judd et al conducted another RCT in 2009. At a 1-year follow-up evaluation, Hagie pins were found to have no advantage over suspension.^[9]^ Despite a number of other randomized controlled trials have been conducted since then, the question is still highly relevant, as the question of whether the surgical treatment is most appropriate for all the patients with displaced fractures of the clavicle is still inconclusive and the reported meta-analysis is ambiguous.^[10–14]^

For this prospective RCT, it is implemented for the comparison of the outcomes of open reduction and internal fixation versus the sling treatment for mid-shaft clavicle fractures. It was hypothesized that in the case of clavicular comminuted mid-shaft fracture, for the open reduction and internal fixation, its nonunion rate was lower and satisfaction rate was higher, although there were a variety of complications.

## 2 Materials and methods

### 2.1 Study design and patients

Approval for this prospective RCT was obtained from the institution alethics review committee of Wuzhong People's Hospital. This trial had been registered with Research Registry, and the number is researchregistry6295. All the operations were conducted via a same surgeon with each patient's informed consent. We will evaluate the eligible patients diagnosed with the mid-shaft clavicle fractures in our hospital from December 2020 to December 2021. The outcomes acquired were reported in accordance with the guidelines of Consolidated Standards of Reporting Trials (CONSORT).

In this trial, the criteria for inclusion included: one third of clavicle fracture with at least 1 axial width displacement (Robinson type 2B2 or 2B1); agreed to take part in our research; over 18 years of age; patients with isolated clavicle fracture. While the criteria for exclusion contained: the medial or lateral segment clavicle fracture; open fracture; injuries related to neurovascular; over 21 days of changes from accident.

### 2.2 Randomization

The patient needs to visit emergency department and wear cuff and collar according to the doctor’ introductions until the subsequent visit fracture clinic. The major investigator would determine the eligible patients at each center and then invited these patients to participate. Through utilizing the computer, all the patients would be randomly divided into non-surgical treatment group or the open reduction and plate fixation group. Prior to the randomization, the patients were stratified with the age and gender, as these 2 factors had formerly been demonstrated to be the bone nonunion predictors.

### 2.3 Operative and nonoperative treatments

All patients in the surgery group received prophylactic antibiotics in accordance with the protocol in our hospital. All patients received general anesthesia, without or with the blockade between scalene. All the surgeries were carried out through the orthopaedic consultant designated in the programme or by a professional registrar/researcher under his supervision. In our investigation, all the patients received standardized treatment. A subclavian incision was made and the skeletal muscle flap was raised from fracture segment. And the fixation was implemented via applying the system of Acumed clavicle fixation consisting of preformed titanium plates. After the wound is closed, place the diseased arm in the arm sling. Pendulum and elbow movements were allowed on the first day after surgery, and subsequent mobilization and rehabilitation programs were the same as in the nonoperative group.

In the sling group, the nurse would inform the surgeon that a closure approach should be carried out, and the patient was taken to the cast chamber, where the patient was offered with the commercial sling for the prevention of elbow. The arm and chest of the patients were wrapped via elastic cotton tape, in order to limit the abduction and abduction of arm. No attempt was made to close the reduction.

### 2.4 Outcome measures

The primary outcome was described as the evidence of nonunion at 1 year, which was defined as the intact bone bridge without X-ray fracture after more than 6 months. The secondary outcomes included the function of arm, overall health, adverse events, the satisfaction related to appearance, and pain. The measurement of function was implemented with Disabilities of the Arm, Shoulder and Hand score, and the constant score. The strength was detected via applying a dynamometer as a part of the constant fraction, a hand-held dynamometer that measured the force the patient could generate against a fixed reaction force. The assessment of results was carried out at 6 weeks, 3 months, and 1 year. The radiography included the 30 degrees caudocephalad radiograph and an anteroposterior taken after the injury and at each examination during follow-up. The assessment of the radiographic images was accomplished with the surgeon.

### 2.5 Statistical analysis

In this present experiment, Statistical Package for the Social Sciences (SPSS) 20.0 was applied to perform statistical analyses. For the continuous variables, they were described with error or mean ± SD. For the evaluation of continuous variables, it was implemented via Kolmogorov–Smirnov normality test. A single factor variance analysis was applied to compare the variables between groups with normal distribution. The discrete numerical variables without exhibiting the normal distribution were analyzed through applying Mann–Whitney *U* variance analysis. With the use of chi-square (*χ*^2^) test and the preparation of crosstabs, the determination for the relationships of categorical variables was performed. When *P* is less than .05, the efficacy is viewed to be statistically significant.

## 3 Discussion

For this prospective RCT, it is implemented for the comparison of the outcomes of open reduction and internal fixation versus the sling treatment for mid-shaft clavicle fractures. It was hypothesized that in the case of clavicular comminuted mid-shaft fracture, for the open reduction and internal fixation, its nonunion rate was lower and satisfaction rate was higher, although there were a variety of complications. Some limitations were included in our study. First, it may be challenging to distinguish between nonunion and union in radiography. Nonunion may be subtle and may be missed due to steel plates. Second, the final assessment conducted by the lead author may result in detection bias. Ultimately, since we did not have the patients in high demand, for instance, the professional athletes, we cannot address the defects and difficulties that disunion or malunion may lead to in the person who is physically demanding.

## Author contributions

**Conceptualization:** Guanggao Li.

**Funding acquisition:** Wencai Su.

**Methodology:** Guanggao Li, Junjie Liao.

**Project administration:** Wencai Su.

**Resources:** Wencai Su.

**Software:** Junjie Liao.

**Supervision:** Wencai Su.

**Writing – original draft:** Guanggao Li.

**Writing – review & editing:** Junjie Liao, Wencai Su.

## Abbreviations

Abbreviation: RCT = randomized controlled study.
